# Supplementary material for: Polymorphisms in Pfkelch13 domains before and after the introduction of artemisinin-based combination therapy in Southwest Nigeria
Source: PLoS One. 2025 Mar 31;20(3):e0316479. doi: 10.1371/journal.pone.0316479 (PMC11957316; doi:10.1371/journal.pone.0316479)
Supplement: Supporting information 2 — (ZIP) [file pone.0316479.s002.zip › 199KN2R_PREMIX_Plate_KELCH2_H09.pdf]

Samples: 12943
Bases: 766
Average spacing: 17

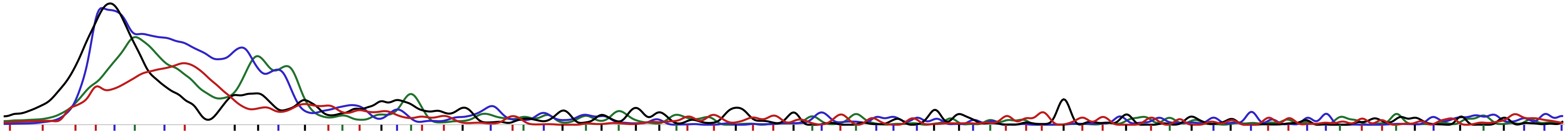

10 20 30 40 50 60 70

T T T T C A C T G G C G T A T G C A T T G C T A C G A G A G G A T T G T T G A C T A C C C G G C A T T T G T T G T A G C G C T T C C A T T A G C G A G C C C

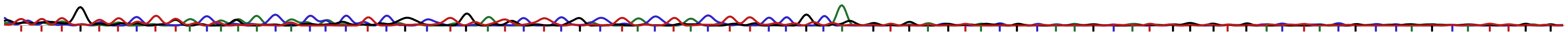

80 90 100 110 120 130 140 150 160

T T T G T T C T T A C A A A C A C C C T C G C T G A T C T C G C T A C T A C T T C C G C A G T G A G T A C G C A A G C A T G G G T G A C T A C G C C A G C A T T G G

170 180 190 200 210 220 230 240

T G A C C A C G C C A A T A T C T C A G A C T A C C T A T C T A A G A A T A T T C T T C C T T G T T T A T C T C T G G G T A C G T G G A G G C C T C C A C T T A A T A

250 260 270 280 290 300 310 320

A T T T C T C T A T A A A T G A A T C T T T T T G G T G T G G T A A G G G A T G G G T A G A T G G T T C A A A A A T A G C T C C A C C G A C A T T A A T A T C A A T C A

330 340 350 360 370 380 390 400 410

T A G T G G C A G T A G C A A T A T T T G G A T C A A C A A T T T T T T T T T A T C A T T C T C A A A A T T T T T A T A T T C T T G A T A A T A T T T T T C T T T T T

420 430 440 450 460 470 480 490

C T A A A T A T A A T A C T A A T T T A A T T T T A T C G A T G T C T T G T A A A A A T C T T A A T C T T T C T T C A T C A A A T C G G G T C C T A T G G G C T T C T

Samples: 12943
Bases: 766
Average spacing: 17

500 510 520 530 540 550 560 570

T T G T G C T T T T G A T T T G G G G G G G C C A T T A G A T A T A T C A A A A T C T A A T T T T C T T T C T T G C T C C A G G G G A T T T C T G T G G C T C C A A

580 590 600 610 620 630 640 650 660

T T T T T T T A C C G G T T T T T T G A T G C T T T T G T A C A A A C C G G G C C C T T T C C C T T T T T T A G A T C T G T T T T G A T C T T A G A A T A A G A T A T C C T G T T

670 680 690 700 710 720 730 740 750

C C T C G A A T A A A A G T C C A T T T G G G G G C T T T T T T G A A C C A A T A A A T G A A G G G T G A G T C T G A A A T C A C G C C A C C A T A G T T T T C A A A A A G C G G C A

760

C C G T C T C T T

Samples: 12943
Bases: 766
Average spacing: 17
